# In Reply: We do not stand a ghost of a chance of detecting plagiarism with ChatGPT employed as a ghost author

**DOI:** 10.3325/cmj.2023.64.293

**Published:** 2023-08

**Authors:** Jan Homolak

**Affiliations:** 1 Department of Pharmacology, University of Zagreb School of Medicine, Zagreb, Croatia; 2 Croatian Institute for Brain Research, University of Zagreb School of Medicine, Zagreb, Croatia *jan.homolak@mef.hr*

I thank Dr Khalifa for his letter to the editor (1) commenting on the article on the adoption of ChatGPT in academic publishing (2). The application of artificial intelligence (AI) and large language models offers the potential to reshape numerous domains of human endeavor, with academia being no exception. Yet, despite the immense promise of these robust tools, their implementation is not devoid of potential pitfalls (3).

Dr Khalifa sheds light on the ethical dilemmas tied to the acknowledgment of ChatGPT use in academic writing. Specifically, he points out that using ChatGPT without openly admitting its involvement could be a form of ghost authorship, with ChatGPT assuming the role of an anonymous (ie, ghost) contributor. At present, the use of ChatGPT and similar tools is not disallowed by publishers, provided they are used solely for enhancing the articulation and linguistic quality of research articles, and this use is explicitly indicated in an appropriate section of the paper.

Nevertheless, while many authors informally reveal their use of ChatGPT to enhance the coherence of their manuscripts, instances of disclosed ChatGPT usage in published papers remain scarce. Authors continue to hesitate when it comes to explicitly acknowledging ChatGPT's contribution, likely due to concerns that such acknowledgment might unfavorably influence the editor's perception of their manuscript, thereby decreasing the chances of their paper being accepted for publication.

The responsibility for adaptation now rests on the shoulders of the publishing system and journal editors, who should encourage authors to acknowledge the legitimate use of ChatGPT. This entails scenarios where authors ensure that the model-modified manuscript remains factually accurate. Among the array of ethical considerations for this, two stand out, both tied to the issue of plagiarism. These aspects underscore the significance of promoting the disclosure of ChatGPT use in manuscript writing, considering both the author's and the publisher's perspectives.

Authors employing ChatGPT as a ghostwriter find themselves in a predicament regarding the potential for inadvertent plagiarism by the tool. Given that the author assumes responsibility for the manuscript, they could be held liable for instances of plagiarism, even if committed by the ghostwriting algorithm.

From the publisher's viewpoint, unreported use of ChatGPT could provide a means for immoral authors to intentionally engage in “plagiarism” without detection. It is crucial to highlight that the interpretation of plagiarism in this context is subject to debate. However, it is also important to recognize that the output produced by ChatGPT can easily evade plagiarism checks, even if the input were classified as entirely plagiarized content.

**Figure 1:**
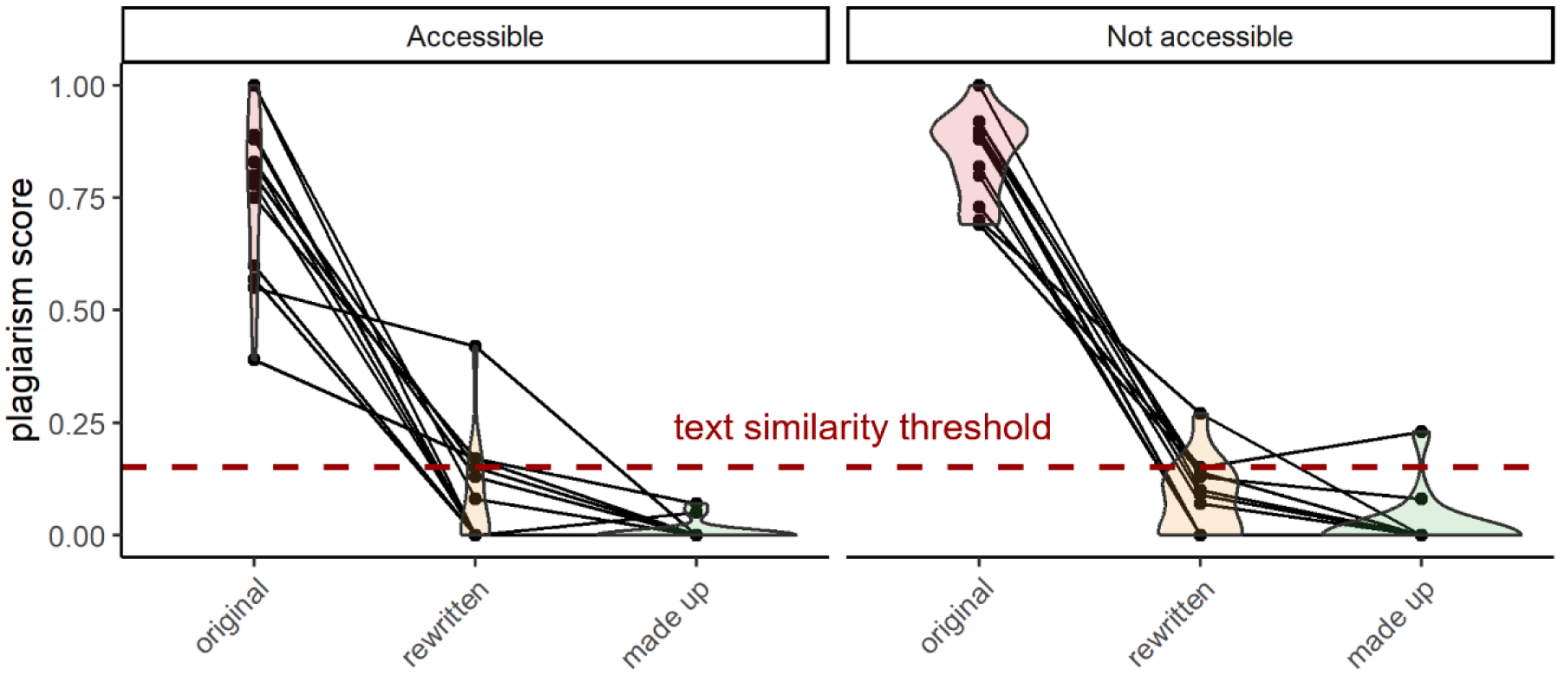
Plagiarism scores for a data set extracted from Homolak (2). This data set comprises 28 original scientific abstracts that were initially composed without the use of ChatGPT (“original”), the same abstracts rephrased by ChatGPT (“rewritten”), and entirely fabricated abstracts created using only the title and authors' names as input for the algorithm. For evaluating text similarity across all abstracts, the freely available Duplichecker plagiarism detection tool was employed. The abstracts (re)written by ChatGPT-3.5 were produced within the confines of its training, as ChatGPT-3.5 does not have the ability to access external sources postdating September 2021. The abstracts were categorized based on whether they were published before (“Accessible”) or after (“Not accessible”) December 2021. In line with convention, a text similarity threshold of 15% was established. As anticipated, most original abstracts scored significantly on the plagiarism scale, serving as true positives due to their existing online publication. Intriguingly, the abstracts rewritten and made-up by ChatGPT yielded notably lower scores compared with the originals. Nevertheless, certain abstracts, especially among those written based on abstracts published before December 2021, still scored relatively well. This suggests that ChatGPT might have incorporated phrases from the initial abstracts, signifying a potential plagiarism attempt. Remarkably, fully fabricated abstracts consistently scored below the threshold, irrespective of their input containing true positives (ie, plagiarized content). This trend persisted for abstracts published after December 2021 as well. A subset of abstracts (re)written by ChatGPT exhibited scores surpassing the plagiarism threshold, a finding hinting at plagiarism attempts potentially detectable by algorithms.

As a demonstration of this concept, a data set consisting of 84 original, rewritten, or entirely fabricated abstracts, sourced from Homolak (2), was analyzed with a plagiarism detection tool (Figure 1). Both the rewritten and fabricated abstracts exhibited remarkably low text similarity scores, even though the input content was entirely sourced from existing publications (considering that all the abstracts are openly accessible and published online). Of significance, instances where rewritten and fabricated manuscripts surpassed the 15% text similarity threshold suggest the potential of ChatGPT to engage in plagiarism (Figure 1).

In conclusion, it is plausible that ChatGPT is being employed as a ghost author. For this reason, publishers and editors should make diligent efforts to guarantee that the use of ChatGPT for enhancing manuscript readability is consistently and prominently disclosed (4). Ensuring that ChatGPT is not used as a ghost author is essential for bolstering the transparency of academic publishing.

**Figure:**
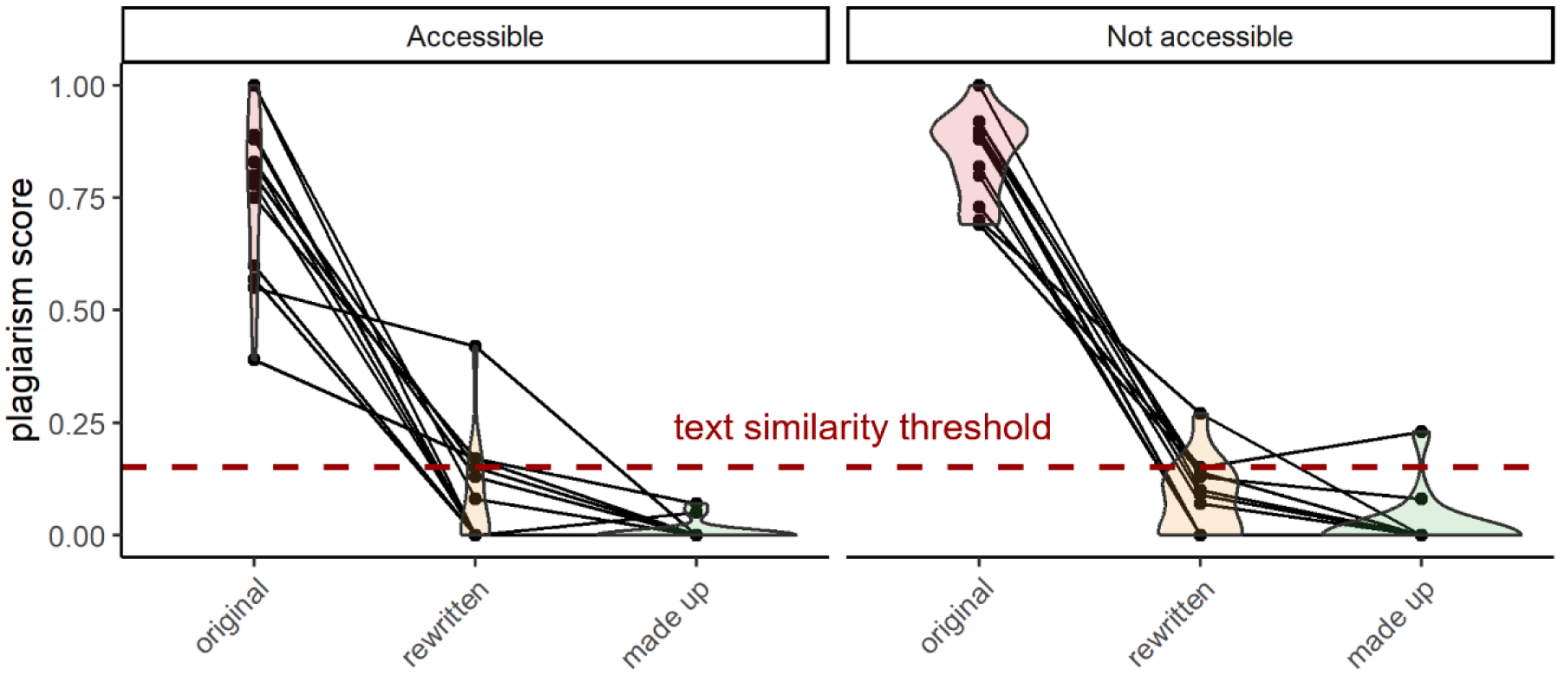
Acknowledgement The manuscript's readability was enhanced using ChatGPT-3.5, with the author's original text serving as the input.
